# Advancements in Computer-Aided Diagnosis of Celiac Disease: A Systematic Review

**DOI:** 10.3390/biomimetics9080493

**Published:** 2024-08-14

**Authors:** Ivana Hartmann Tolić, Marija Habijan, Irena Galić, Emmanuel Karlo Nyarko

**Affiliations:** Faculty of Electrical Engineering, Computer Science and Information Technology, J. J. Strossmayer University, 31000 Osijek, Croatia; marija.habijan@ferit.hr (M.H.); irena.galic@ferit.hr (I.G.); karlo.nyarko@ferit.hr (E.K.N.)

**Keywords:** artificial intelligence, celiac disease, computer-aided diagnosis, computer vision, machine learning

## Abstract

Celiac disease, a chronic autoimmune condition, manifests in those genetically prone to it through damage to the small intestine upon gluten consumption. This condition is estimated to affect approximately one in every hundred individuals worldwide, though it often goes undiagnosed. The early and accurate diagnosis of celiac disease (CD) is critical to preventing severe health complications, with computer-aided diagnostic approaches showing significant promise. However, there is a shortage of review literature that encapsulates the field’s current state and offers a perspective on future advancements. Therefore, this review critically assesses the literature on the role of imaging techniques, biomarker analysis, and computer models in improving CD diagnosis. We highlight the diagnostic strengths of advanced imaging and the non-invasive appeal of biomarker analyses, while also addressing ongoing challenges in standardization and integration into clinical practice. Our analysis stresses the importance of computer-aided diagnostics in fast-tracking the diagnosis of CD, highlighting the necessity for ongoing research to refine these approaches for effective implementation in clinical settings. Future research in the field will focus on standardizing CAD protocols for broader clinical use and exploring the integration of genetic and protein data to enhance early detection and personalize treatment strategies. These advancements promise significant improvements in patient outcomes and broader implications for managing autoimmune diseases.

## 1. Introduction

Celiac disease (CD) is a complex autoimmune disorder characterized by an abnormal immune response to dietary gluten, leading to inflammation and damage to the small intestine in genetically predisposed individuals. It is estimated that nearly 1% of the global population is affected by this condition, making it a prevalent health concern worldwide [1]. Accurate and timely diagnosis of CD is of paramount importance for individuals suffering from the condition, as untreated CD can lead to a range of severe health complications, including malnutrition, osteoporosis, and an increased risk of certain malignancies [2].

Biomimetics, the application of principles and designs found in nature to engineering and technology, offers a promising avenue for developing intelligent diagnostic tools for CD. By emulating biological processes, such as the immune system’s response to gluten, researchers are developing computer-aided diagnostic (CAD) systems that can analyze complex datasets, including genetic markers, serological data, and clinical symptoms, to provide more accurate and timely diagnoses. These bio-inspired approaches have the potential to revolutionize CD diagnosis by offering non-invasive, efficient, and personalized diagnostic tools.

Traditionally, the diagnosis of CD has relied on a combination of clinical symptoms, serological tests, and histopathological examination of intestinal biopsy samples. While this approach has been effective, its limitation is in the variability of clinical presentation and the invasiveness of biopsy procedures [3]. Moreover, the evolving understanding of the disease has revealed a spectrum of CD presentations, including non-classical and asymptomatic forms, further complicating diagnosis. Therefore, traditional diagnostic methods often require invasive procedures and are prone to subjectivity.

Recently, there has been a growing interest in harnessing the power of advanced technologies to improve the accuracy and efficiency of CD diagnosis [1]. Computer-aided diagnosis (CAD) systems, often utilizing artificial intelligence (AI) and machine learning (ML) algorithms, have emerged as promising tools in this context. These systems have the potential to analyze complex datasets, including genetic markers, serological data, and clinical symptoms, and provide more accurate and timely diagnoses. While some methods are promising, the road to establishing computer-aided CD diagnosis as a reliable diagnostic tool is still long, necessitating further research, validation, and integration into clinical practices.

The primary motivation behind this systematic review is to evaluate the recent advancements in CAD techniques for CD diagnosis. The primary aim is to show that computer-aided celiac disease diagnosis is achievable without requiring complex and resource-intensive algorithms, potentially making diagnosis more accessible and efficient [4]. The objectives of this study are as follows:

- To provide a systematic overview of CAD-based methods for CD diagnosis;
- To highlight the strengths and limitations of current methods in order to give guidance for improving the quality of future research;
- To provide a discussion of the existing challenges of commonly applied techniques to identify gaps in the existing research and highlight areas where further investigation is needed.

By synthesizing and analyzing the existing literature, we seek to evaluate the current state of CAD systems, their diagnostic accuracy, and their potential use in clinical settings. We anticipate that our findings will serve as a comprehensive guideline and a catalyst for future research directions and the development of more sophisticated algorithms.

### Review Methodology

In February 2024, we conducted a comprehensive search for relevant literature across several prestigious online databases, including, but not limited to, the databases shown in Table 1. This search was guided by topics (TS) related to our focused query: ((TS = (computer aided OR cad OR deep learning OR machine learning OR artificial intelligence)) AND TS = (celiac disease OR celiac sprue)) AND TS = (segmentation OR detection OR classification OR identification OR evaluation OR diagnosis).

**Figure 1:**
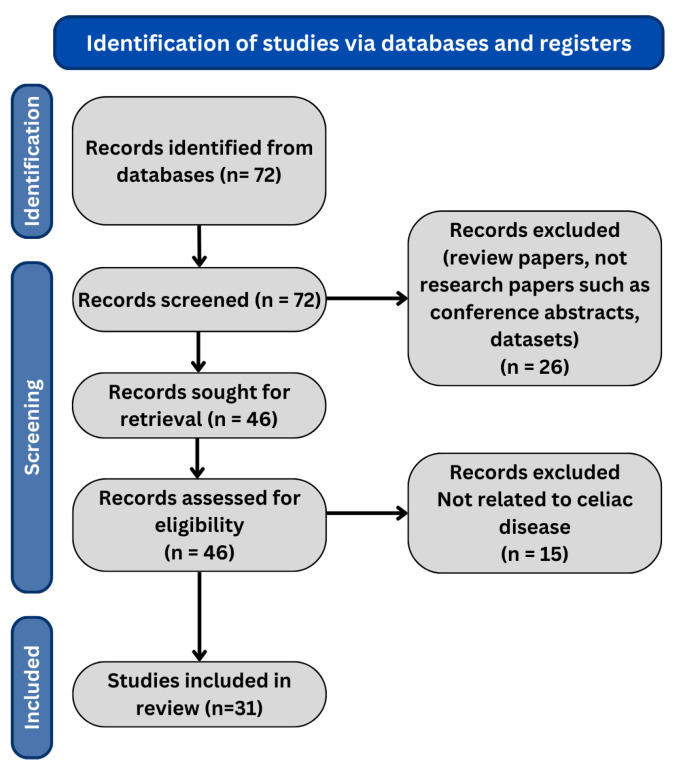
PRISMA flow diagram of our review methodology.

**Figure 2:**
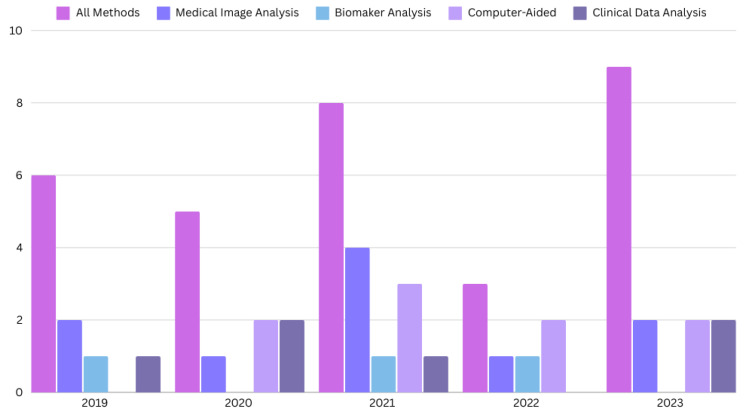
The distribution of the papers for CD diagnosis published in the last five years.

The search for the last 5 years resulted in 67 research papers in total. We only include the journal and conference research papers (case reports, case series, preclinical studies, and reviews are excluded). The PRISMA flow diagram is shown in Figure 1, and the distribution of research papers per year is shown in Figure 2.

## 2. Clinical Background

**Figure 3:**
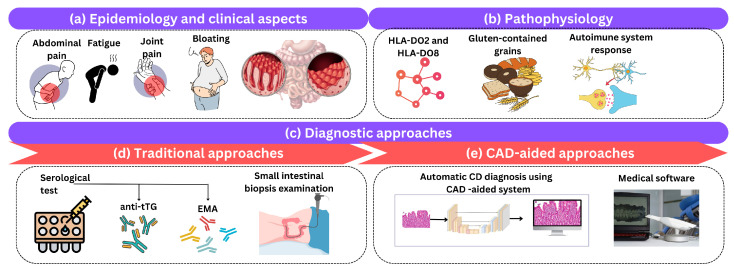
An illustration of (**a**) epidemiology and clinical aspects, (**b**) pathophysiology, and (**c**) diagnostic approaches of CD. Diagnostic approaches include (**d**) traditional and (**e**) computer-aided approaches.

Celiac disease (CD), also known as gluten-sensitive enteropathy or celiac disease, is a long-term autoimmune condition that primarily affects the small intestine and is triggered by the consumption of gluten-containing grains. This section briefly reviews the clinical aspects of CD, its epidemiology, pathophysiology, and the traditional and CAD diagnostic methods used in clinical practice as shown in Figure 3.

### 2.1. Celiac Disease Epidemiology and Clinical Aspects

CD manifests with a range of clinical aspects that span gastrointestinal and extra-intestinal symptoms. Gastrointestinal symptoms often include diarrhea, abdominal pain, bloating, and weight loss [5]. However, CD is a systemic disorder, and extra-intestinal symptoms such as fatigue, joint pain, and dermatitis herpetiformis may also be present. The clinical presentation can widely vary among individuals, making the diagnosis challenging, and highlighting the importance of considering both gastrointestinal and extra-intestinal symptoms when evaluating patients for CD [6]. Recently, it has undergone a significant shift in epidemiological understanding over the years. Once perceived as a rare condition, it is now recognized as a prevalent autoimmune disorder affecting approximately 1% of the general population [1]. The prevalence varies across different regions and ethnic groups, emphasizing the influence of genetic factors. Familial clustering is common, with first-degree relatives of individuals with CD having an increased risk [7]. This evolving epidemiological landscape underscores the necessity for heightened awareness and improved diagnostic strategies in primary care settings. It also points to the critical role of genetic and environmental research in unraveling the complex etiology of CD, guiding more personalized and effective treatment approaches.

### 2.2. Celiac Disease Pathophysiology

The pathophysiology of CD unveils a complex interplay between genetic susceptibility, environmental triggers, and the immune system. Individuals carrying specific human leukocyte antigen (HLA) types [8], particularly HLA-DQ2 and HLA-DQ8, are genetically predisposed to CD [9]. The key environmental trigger is the consumption of gluten-containing grains, such as wheat, barley, and rye. Upon gluten ingestion, a cascade of events is set in motion, involving the immune system’s inappropriate response [10]. Gluten peptides initiate an immune reaction in the small intestine, leading to the activation of T cells and the production of antibodies, notably anti-tissue transglutaminase (anti-tTG) and anti-endomysial antibodies (anti-EMA). This autoimmune response results in chronic inflammation, causing damage to the lining of the small intestine. Villous atrophy (VA) and crypt hyperplasia are characteristic histological features [11], impairing nutrient absorption and contributing to the diverse clinical manifestations of CD. The intricate pathophysiology underscores the need for a multi-faceted approach to diagnosis and management, taking into account both genetic predisposition and environmental factors.

### 2.3. Bio-Inspired Intelligent Approaches for CD Diagnosis

Bio-inspired intelligent methods integrate phenomena into computer-aided design (CAD) systems, improving the accuracy and efficiency of CAD systems in the diagnosis of diseases. The evolutionary algorithm (EA) is another approach to refining algorithms and draws inspiration from selection. EAs can adapt machine learning models to better distinguish between individuals with disease and healthy individuals. By mutating and crossing model parameters, EAs can discover solutions that are difficult to achieve through parameter adjustments. Swarm intelligence (SI) algorithms study the behavior of organisms such as ants and bees to make informed decisions. These algorithms help select biomarkers or clinical variables that are critical for diagnosis by efficiently exploring a variety of traits and converging to optimal solutions by simulating swarm interactions and decision-making processes.

Traditional diagnosis of the disease typically entails evaluation, serologic testing, and confirmation via small bowel biopsy [12]. It serves as a screening method for tests such as anti-tTG and anti-EMA that detect antibodies. This diagnostic approach is vital for evaluating small bowel biopsies to assess the degree of atrophy (VA) and other histopathological changes. In the past, this approach has shown success. Ongoing research is aiming to enhance its precision and effectiveness.

The analysis of complex datasets, possible biomarkers, and higher diagnostic accuracy are some of the areas where ML algorithms are promising. These tools contribute to better diagnostic accuracy. Physicians can use computational techniques to interpret the results of histopathology examinations, especially for small bowel biopsies. Integrated into healthcare systems, these technologies could transform diagnosis. They can improve effectiveness. This can lead to personalized care for CD patients. The most common technology used in the diagnosis of CD is artificial neural networks. ANNs mimic the neurons found in biological systems. These networks can be trained on large datasets of medical images, biomarkers and clinical data to recognize patterns indicative of CD. For example, Convolutional Neural Networks (CNNs), a special type of ANN, have shown remarkable success in analyzing endoscopic images and histopathological specimen to detect characteristic features of CD, such as villous atrophy.

## 3. Celiac Disease Diagnostic Methods

There are several methods in the field of computer-aided diagnosis of CD, including the analysis of medical images, the use of biomarkers and the development of computer-aided models. Each of these approaches offers different diagnostic features and characteristics that contribute to a comprehensive assessment of CD.

However, it must be acknowledged that the uniqueness of the methods used in CD diagnosis poses certain challenges. Authors often use similar or identical methods and algorithms in their research, especially in the context of diagnostic techniques such as endoscopy with biopsy, image processing and analysis, blood testing, genetic data analysis, machine learning (ML), deep learning (DL), and clinical data analysis.

Despite the overlap in the methods used by researchers, these methods work together to improve diagnostic accuracy. The integration of different techniques facilitates a holistic understanding of CD presentation in patients and emphasizes the importance of combining approaches for solid diagnostic results.

This methodological breakdown serves to highlight the multi-faceted nature of CD diagnosis and provide a comprehensive understanding of different approaches used to achieve accurate diagnostic results in CD patients. By combining techniques, a comprehensive understanding of the presence of CD in a patient is gained.

Each of these methods has specific diagnostic features and characteristics as described below:

- Medical image analysis:
  - Endoscopy with biopsy: computer techniques can be used to analyze endoscopic images of the small intestinal mucosa to identify changes characteristic of CD.
  - Image processing and analysis: image processing algorithms can help identify morphological changes in intestinal tissue associated with CD.
- Biomarker analysis:
  - Blood tests: computational methods can be used to analyze laboratory results, including the presence of specific antibodies (such as anti-tTG antibodies) indicative of CD.
  - Analysis of genetic data: computer methods can be used to analyze genetic markers associated with CD to determine genetic predisposition.
- Computer-aided models:
  - ML: enables the development of models that can be trained on datasets to identify characteristic patterns of CD based on medical images or clinical data.
  - DL: is a branch of ML that uses deep neural networks to process complex data. It can be applied to the analysis of medical images or genetic data to diagnose CD.
- Clinical data analysis:
  - Symptom data: computational methods can analyze patient’s symptoms and clinical data to identify patterns suggestive of CD.

**Figure 4:**
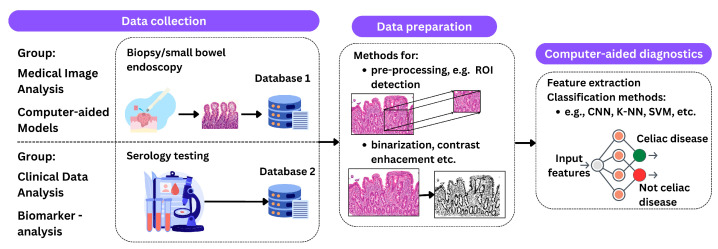
An illustration of the described processing methods. The process begins with data acquisition, which includes procedures such as biopsy and endoscopy for medical imaging and serologic testing for clinical data analysis. The next phase, data preparation, includes techniques such as ROI detection, contrast enhancement, and binarization. The final step is classification using methods such as CNNs, K-NNs, and SVMs to predict the presence of celiac disease.

Based on the literature reviewed, it is concluded that medical images, biomarkers, and clinical records are used to make a final decision on the positive or negative outcome of testing for celiac disease, mainly using machine learning and neural networks as shown in Figure 4.

In medical image analysis, the field that deals with clinical challenges by examining images from the clinical environment, deep learning techniques are used, with deep convolutional networks playing an important role. With the proliferation of digital clinical images, there is an urgent need for methods that are optimized for big data analysis. Medical imaging involves processes that aim to visualize the human body, which is crucial for improving diagnostic and treatment procedures. Medical imaging includes various imaging modalities such as X-ray, CT, MRI, PET, and ultrasound, and plays a central role in the diagnosis and treatment of diseases, increasing the efficiency of radiologists and clinicians [13]. These processes can be used for image processing algorithms to detect damage to the small bowel or other characteristic features that may appear in medical images.

On the other hand, CAD refers to the development of computer models, such as methods of data analysis, including machine learning and neural networks, to facilitate the diagnosis of celiac disease. These models can use various inputs, including medical images, biomarkers, and genetic data to generate diagnostic results or predict the positive or negative outcomes of testing for celiac disease. Traditional approaches in computer-aided diagnosis (CAD) for medical imaging use image analysis to recognize disease patterns and distinguish between different classes of structures on images, e.g., normal versus abnormal or malignant versus benign. CAD developers design image processing and feature extraction methods based on expert knowledge to capture image features that distinguish between different conditions. The extracted features serve as input prediction variables for a classifier that forms a predictive model by adjusting feature weights based on the statistical properties of a training sample to estimate the probability that an image belongs to a particular state [14].

### 3.1. Medical Image Analysis

Feature extraction plays a central role in the detection and classification of CD. It involves the exploration of various pattern recognition methods to effectively classify the extracted features in the field of medical image analysis and ensure that they are assigned to the correct class [15].

Hegenbart et al. [16] proposed a computer-aided diagnosis of CD using a scale-invariant texture classification. Gastroscopic visuals provide a diverse representation of the mucosal surface, varying in texture and scale based on the camera’s angle and proximity to the mucosal lining. The authors’ work aims to provide a general scale-invariant texture descriptor for the classification of duodenal mucosa texture. They proposed a new affine invariant method based on Local Ternary Patterns (LTPs). According to the grading scheme of the mucosal condition, they divided the available duodenal images into four different classes (normal crypts and villi and three types of classes of crypts and villi, i.e., the characteristic changes caused by CD). They proposed several methods to ensure scale invariance to determine that scale invariance improves results in detecting CD. Findings from employing a k-NN classifier indicate that scale invariance does not play a crucial role in the classification of CD across two distinct databases.

Kowsari et al. [17] proposed a unique approach to medical image classification that departs from the standard multi-class classification paradigm. Instead, it employs a Hierarchical Medical Image Classification (HMIC) method. HMIC comprises a hierarchy of DL models that provide discriminative insights at different levels of clinical image analysis. In their experiments, they applied the HMIC approach to assess small bowel enteropathies, specifically to distinguish between environmental enteropathy (EE), CD, and histologically normal controls. Since biopsy images are often large, unstructured, and high resolution, the authors divided them into smaller areas to allow processing by a deep neural network. The obtained results indicate that the HMIC approach, which includes multiple levels of Convolutional Neural Networks (CNNs), provided robust results at both higher and lower classification levels. Importantly, the accuracy achieved outperformed traditional methods such as CNN, multi-layer perceptrons, and DCNN. This highlights the potential of hierarchical deep learning to improve classification and provides a flexible approach to analyzing medical data within a hierarchical framework that goes beyond traditional multi-class classification methods.

Saken et al. [15] introduced a hybrid algorithm that leverages ML techniques for the automated identification of CD through standard endoscopic procedures. An analysis comparing non-segmented and segmented images showed that the context-based image segmentation method consistently achieved higher accuracy. The goal is to develop a comprehensive CAD system that integrates various image processing methods, including segmentation, feature extraction/selection, and finally classification. Their proposed algorithms have shown high efficiency in the automatic diagnosis of CD.

The study by Syed et al. [18] tackled the complex issue of diagnosing diseases with overlapping histopathological characteristics, focusing specifically on small bowel enteropathies (EE and CD). These conditions present a critical clinical challenge, necessitating the creation of computational techniques for precise and quantitative assessment. To address this, the researchers unveiled an AI-driven platform for image analysis, utilizing DL through CNNs to differentiate between these diseases effectively. The collection of data spanned three distinct studies across various locations. This AI platform integrates a multi-scale architecture and employs gradient-weighted Class Activation Maps (grad-CAMs) to visually represent the model’s decision-making process. Medical experts conducted evaluations on the images to detect any biases and carefully examined the grad-CAMs to ensure that the structural integrity and biomedical relevance were maintained. The dataset comprises 461 high-resolution biopsy images from 150 pediatric patients, with an average age of 37.5 months and a balanced gender ratio. Performance evaluations revealed that the ResNet50 and Shallow CNN models achieved case detection accuracies of 98% and 96%, respectively. This accuracy further improved to 98.3% when the models were combined into an ensemble approach. The grad-CAMs effectively showcased the models’ capability to discern the distinct microscopic morphological characteristics of EE, CD, and control samples. These findings underscore the significant potential of AI to enhance the diagnosis of diseases with histopathological similarities.

Moreover, in their other work, Syed et al. [19] introduced a DL-based image analysis platform designed for the automatic extraction of quantitative morphological features from gastrointestinal biopsy images. The objective of this platform is to pinpoint distinctive features capable of differentiating between similar conditions, particularly between EE and CD, within the pediatric malnutrition context. The aim is to forge data science techniques that merge histopathological, clinical, and molecular data to create prognostic models that are both insightful and clinically applicable. The findings reveal that this AI-driven histopathological evaluation framework achieves a remarkable 93.4% accuracy rate in classifying and distinguishing duodenal biopsies from children diagnosed with EE and CD, showcasing its significant diagnostic capabilities.

Faust et al. [20] investigated whether AI models can help distinguish between healthy individuals, individuals with CD, and individuals with non-celiac disease (NCD) based on features of the lamina propria of the small intestine (LP). The research presents a novel ML algorithm for the automated analysis of small intestine biopsy images that specifically focuses on LP inflammatory cells in the duodenum for normal, CD and NCD cases. The main objective is to investigate seven different scenarios that vary the composition of LP cells to find the classification that achieves the highest accuracy among the proposed classifiers. The dataset includes duodenal biopsy images from individuals without bowel disease, CD, and NCD patients. The results show that the Support Vector Machine (SVM) achieves 98.53% accuracy in discriminating between normal controls and CD and 98.55% in discriminating between normal controls and NCD when a linear kernel is used. These results suggest the potential of AI to support the automated analysis of small intestinal biopsies for the assessment of villous architecture is challenging.

Sali et al. [21] explored the severity assessment of CD by employing CNNs to analyze histopathological images. Their innovative approach involved training a model to differentiate among various stains in whole slide images (WSIs) and devising a strategy to aggregate stain classifications to deduce insights on entire WSIs. Remarkably, their model achieved flawless classification accuracy at the WSI level. The validation outcomes are highly encouraging, indicating the model’s capability to support pathologists in assessing CD severity through histological evaluations. Furthermore, they implemented the grad-CAM technique to visually elucidate microscopic features that signify the severity of CD, enhancing the understanding and interpretability of the diagnostic process.

The study by DiPalma et al. [22] introduced an innovative DL methodology aimed at enhancing the computational efficiency of classifying histology images for CD. This model surpasses a high-resolution teacher model in crucial metrics such as accuracy, F1 score, precision, and recall, all the while requiring substantially less computational power—up to four times less. The research strategy focuses on reducing computational demands without compromising on classification accuracy. Additionally, the study underscores the advantage of incorporating unlabeled data, which notably boosts the performance of CD classification, particularly at lower magnifications. Furthermore, the proposed method facilitates the analysis of WSI at significantly reduced resolutions, without a notable decrease in classification accuracy, demonstrating a promising avenue for efficient and accurate disease diagnosis.

Garbaz et al. [23] explored the application of capsule endoscopy for detecting gastrointestinal bleeding, a pivotal tool for diagnosing various conditions such as gastrointestinal bleeding, early-stage cancer, abdominal pain, Crohn’s disease, CD, polyps, and ulcers. They introduced a novel detection method for bleeding in wireless capsule endoscopy (WCE) images, employing a sophisticated deep neural network. This network synergizes a high-level Inception-ResNet V2 model with a foundational CNN to enhance classification efficacy. Their method achieved an outstanding average accuracy of 98.5%, alongside remarkable sensitivity, specificity, and precision rates of 98.5%, 99%, and 98.5%, respectively. These results underscore the method’s high effectiveness in precisely detecting bleeding within WCE images, which is a critical advancement for the diagnosis and management of gastrointestinal ailments.

Elmes et al. [24] proposed an automatic annotator for medical image analysis. This system learns from a small set of expert annotations collected by tracking the attention of pathologists. It was designed to label new incoming samples for classification and segmentation tasks. The goal was to classify images into five severity levels of CD. They trained their classifier using ResNet-18 and found that accuracy improved significantly when using a dataset with heatmap-based villus recognition, indicating the potential of this method for various medical imaging tasks beyond CD.

Furthermore, the work proposed by Tyagi et al. [25] introduced Deep Guided Posterior Regularization (DEGPR), a method to improve object detectors by guiding them to use discriminative features between cells that can either be provided by pathologists or inferred from visual data. DEGPR was validated on three datasets, including a novel dataset called MuCeD, which comprises 55 biopsy images for the prediction of celiac disease. It includes additional regularization constraints to make the model output posterior distributions of features over predicted bounding boxes similar to ground truth. In experiments with publicly available datasets, DEGPR showed significant improvements in detection and counting, with the model’s F-score increasing from 77% to 90% when predicting CD.

The summary of the methods examined in the available literature based on medical image analysis is shown in Table 2.

### 3.2. Biomarker Analysis

The work by Caetano dos Santos et al. [26] investigates the feasibility of using ML to develop an unbiased and automated method to evaluate and classify the EmA test in the context of CD diagnosis. In traditional classification performed by highly trained professionals, samples are classified into four classes: positive, negative, IgA-deficient, and equivocal. This study represents the first attempt to use ML to automatically classify the IgA class EmA test in CD diagnosis. It demonstrates that ML can enable the rapid and accurate analysis of EmA tests, which has the potential for further streamlining. ML was used to build a classification model that achieved a sensitivity of 82.84% and a specificity of 99.40%, for an overall accuracy of 96.80%.

The study of Magazzù et al. [27], which was part of the ITAMA project, aimed at improving CD diagnosis and closing diagnostic gaps, especially in underdiagnosed cases. The goal was to identify individuals with disease who might benefit from a gluten-free diet, even if they do not have typical gastrointestinal symptoms, abnormal serology, or histologic evidence of celiac disease (CD). This was performed by detecting TGA-IgA mucosal deposits in duodenal biopsies. Point-of-care testing (POCT) and conventional serology were used to diagnose CD. The results were anonymously recorded in a database along with the questionnaire responses. In addition, the study explored the potential of using the first-degree relatives of CD patients as markers for CD by analyzing the presence of anti-tTG-2 mucosal deposits. The development of an AI-based clinical decision support system (CDSS) was another important outcome of the ITAMA project. This CDSS, based on a fuzzy neural network classifier, was trained and tested on both simulated and real data to improve the diagnosis of CD by identifying optimal separation scenarios between positive and negative cases.

Shemesh et al. [28] explored the distinction in naive B-cell receptor (BCR) repertoires between individuals with CD and healthy subjects, employing ML techniques for data analysis. The investigation effectively differentiated these clinical cohorts and pinpointed specific patterns that could act as markers for CD. Notably, it was revealed that patients with CD exhibit groups of BCRs that are uniquely characterized in comparison to those of healthy controls, highlighting the possibility of using these findings for the early identification of individuals at risk for CD. This also sheds light on the immunological mechanisms underlying CD and autoimmunity at large. Traditional metrics for classifying BCR repertoires, such as the utilization of particular BCR genes, sequence lengths, and isotypes, showed no significant differences between the CD patients and healthy participants. However, unique clusters of shared motifs were instrumental in distinguishing CD patients from healthy subjects, achieving an F1 score of 85% in classification accuracy using naive BCR repertoires from combined IgH and IgL sequences.

The summary of the methods examined in the available literature based on biomaker analysis is shown in Table 3.

### 3.3. Computer-Aided Models

The study proposed by Wang et al. [29] introduced and examined a cutting-edge block-wise channel squeeze-and-excitation (BCSE) learning module, designed to elevate the efficacy of deep learning networks in detecting CD. This module was effortlessly incorporated into the architectures of ResNet50 and Inception-v3 to augment their detection capabilities. Through preprocessing of the initial images and feature extraction via convolutional blocks, the BCSE module recalibrates the importance of channel information on a block-wise basis, focusing on locally prominent features. Furthermore, the squeeze-and-excitation (SE) block plays a crucial role in adaptively fine-tuning the features of CD images, thereby amplifying critical pathological details while minimizing irrelevant information. Additionally, the integration of ResNet50 with an SVM classifier proved to be highly effective in identifying minor VA characteristics of celiac disease, underscoring the significant potential of the BCSE module to enhance the accuracy of CD diagnosis within deep learning frameworks.

Khan et al. [30] investigated duodenal and colonic samples from children diagnosed with Environmental Enteric Dysfunction (EED), CD, and various other gastrointestinal conditions. They employed a combination of quantitative mucosal morphometry, histopathological evaluation, and advanced image analysis bolstered by ML techniques, analyzing cohorts from both Pakistan and the United States. A notable finding was the more severe villus blunting in CD compared to EED, particularly among Pakistani patients. Utilizing the ResNet50 model for ML-powered image analysis, the study discerned varying degrees of accuracy in classifying disease types based on biopsy samples from duodenal and rectal tissues. It was observed that analyses of duodenal tissues were less accurate than those of rectal tissues, with certain predictive overlaps noted across different conditions. Furthermore, Grad-CAM saliency maps were instrumental in identifying specific characteristics unique to celiac disease and chronic duodenitis, shedding light on the morphological similarities present among children from regions with limited resources, diagnosed with EED, chronic duodenitis, and CD.

Koh et al. [31] introduced a CAD system specifically engineered to address the diagnostic complexities associated with identifying CD. This innovative system employs discrete wavelet transform (DWT) to decompose video images, facilitating the extraction of both textural and non-linear characteristics. Using particle swarm optimization (PSO), the system selects 30 optimal features for classification. Using 10-fold cross-validation, it achieves an impressive 86.47% accuracy, as well as high sensitivity (88.43%) and specificity (84.60%). Using the leave-one-out cross-validation (LOOCV) technique, it also achieves an accuracy of 85.91%. This CAD system holds significant potential for improving celiac disease detection and could reduce misdiagnosis. The method involves feature extraction from video capsule images and could find application in the clinical setting to assist physicians in accurate diagnosis and timely treatment.

In the study proposed by Molder et al. [32], endoscopic images from the duodenum of individuals newly diagnosed with CD and non-CD controls were gathered and subjected to analysis using ML and DL techniques to identify VA. The algorithms tested demonstrated impressive diagnostic precision, with a multi-layer CNN achieving the highest performance, marked by a sensitivity of 99.67% and a positive predictive value of 98.07%. This initial study introduced a highly accurate, automated method for identifying mucosal alterations indicative of VA in CD patients, distinguishing these from the normal, non-atrophic mucosa observed in non-CD controls, using histology as the benchmark. The participants were individuals with clinical suspicion of CD who had undergone serological tests and upper gastrointestinal endoscopy, including multiple biopsies from the duodenum. The findings indicate that the computerized identification of VA through endoscopy image analysis is a viable approach and could act as an effective method for CD case identification. The layered CNN algorithm, in particular, showed exceptional sensitivity (99.30%) and a positive predictive value of 98.61% when analyzing image segments from the duodenum of patients newly diagnosed with CD. While ML techniques yielded strong results, DL methods, especially those involving CNNs, provided marginally superior outcomes.

Vicnesh et al. [33] introduced a novel method for feature extraction employing DAISY descriptors to evaluate CD in video capsule endoscopy (VCE) images. By preprocessing the images to eliminate edges, the advanced capabilities of the DAISY descriptor were leveraged for the efficient extraction and classification of features indicative of CD. This technique showed significant effectiveness in isolating pertinent characteristics from small bowel VCE images. Further refinement was achieved through the application of Shannon entropy for feature reduction and particle swarm optimization (PSO) for the crucial step of feature selection, enhancing the classification process. Traditional diagnostic procedures for CD tend to be time-consuming, manual, and expensive. In response, a CAD system utilizing DAISY descriptors was developed, automating the diagnosis of celiac disease. This system demonstrated a promising accuracy rate of 89.82% with ten-fold cross-validation, showcasing the potential for automated diagnostic solutions in the field of CD.

Stoleru et al. [4] proposed an innovative approach that uses special filters to identify specific artifacts in endoscopy images and then applies classified ML techniques. These artifacts include mucosal atrophies with visible submucosal vascular patterns, crack-like depressions, reduced or absent duodenal folds, submerged appearances at Kerckring folds, and low numbers of villi. The novelty of this method lies in the approach that includes two modified filters, including one specifically designed for these images. After applying the filters, the algorithm adjusts the images to analyze the texture of the intestinal wall, eliminating the need for complex ML algorithms. For an accurate diagnosis, the system requires five images from specific regions of each patient’s gastrointestinal tract. Instead of complex learning algorithms, the system relies on identifying specific artifacts in endoscopy images using filters and then training a classified machine learning algorithm. In this study, the goal was to classify patients as either healthy or having CD based on data from the camera of an endoscopic device. Three algorithms were used for this learning task. The weighted KNN algorithm achieved 92.2% accuracy, with only 4 out of 51 tests giving false results, while the linear SVM algorithm achieved 94.1% accuracy, with 3 out of 51 tests giving false results.

Zammit et al. [34] introduced a method to study the total CD burden in the small intestine. The study aimed to quantitatively assess the severity of CD using ML algorithms and compare their performance with that of three experienced gastroenterologists. They investigated potential associations between the VCE results and clinical parameters such as the physician’s overall assessment, serology, and biopsy findings. The study highlighted the potential of VCE and AI tools to transform subjective tests into quantitative and reproducible measurement tools for CD diagnosis and monitoring. The proposed method demonstrated effective performance in grading the severity of CD, and their scores showed a strong correlation with those of human experts, indicating their reliability. Therefore, this research focused on the objective assessment of villous damage in CD patients by human experts using an unbiased VCE assessment tool and ML-based disease assessment.

Koh et al. [35] introduced an ML approach designed to automate the analysis of biopsy images for the detection and classification of VA, a crucial marker in diagnosing CD according to the modified Marsh score. It stands out as one of the pioneering efforts to apply traditional ML techniques for automating the assessment of biopsy images in identifying and categorizing CD. Utilizing the Steerable Pyramid Transform (SPT), the method captures sub-bands, from which it extracts a variety of entropy and non-linear characteristics. The findings demonstrate noteworthy accuracy levels, including an 88.89% success rate for distinguishing two-class villi anomalies in hematoxylin and eosin (H&E) stained biopsy images, 82.92% for two-class differentiation in red-green-blue (RGB) biopsy images, and 72% for multi-class classification. This technique offers a significant opportunity to enhance both the efficiency and precision of CD diagnoses through the automated analysis of biopsy images.

In the study proposed by Scheppach et al. [36], the researchers compiled a dataset of endoscopic images to train a deep learning model, specifically ResNet18, for the identification of VA. Upon applying this AI algorithm to an external dataset, it was found to achieve a sensitivity of 90%, a specificity of 76%, and an overall accuracy of 84%. This performance surpassed that of both endoscopy fellows and experts, marking a significant enhancement in diagnostic capabilities for endoscopists lacking specialized expertise. Notably, the AI algorithm’s effectiveness remained consistent across challenging images, showcasing its potential as a reliable decision-support tool for detecting VA in endoscopic imagery.

In the study proposed by Wimmer et al. [37], the use of image-to-image translation for conversion between conventional white-light imaging (WLI) and narrow-band imaging (NBI) in endoscopic images for celiac disease diagnosis was investigated. The methods used in this study involve the use of Convolutional Neural Networks (CNNs). The effectiveness of training models on virtual or mixed virtual-real data to improve classification accuracy on limited labeled data, and whether translating test images to a different domain increases accuracy, was investigated. The results indicated successful conversion between WLI and NBI, improved classification with virtual training data, and slightly increased accuracy when converting test images, suggesting potential benefits for celiac disease diagnosis.

In the study proposed by Maleki et al. [38], a deep learning-based pipeline for the diagnosis of celiac disease (CD) using whole slide images of intestinal biopsies was presented. The pipeline showed superior performance compared to typical supervised deep learning approaches, achieving precision, recall, and accuracy of 0.941, 0.889, and 0.893, respectively, in discriminating celiac disease from normal tissue. Despite its effectiveness, limitations arose due to the challenge of obtaining a sufficient number of samples for training deep learning models, especially for whole slide images. Although the pipeline focuses on CD diagnosis, it can be adapted to various histopathology image classification tasks, offering potential utility in clinical contexts where large pathology datasets are impractical.

The summary of the methods examined in the available literature based on computer-aided models is shown in Table 4.

### 3.4. Clinical Data Analysis

Tomer et al. [39] developed a computational methodology to forecast epitopes and motifs linked with CD, utilizing a dataset comprising both experimentally verified CD-related and non-CD-related peptides for the purposes of training, testing, and assessment. The investigation involved analyzing the positioning of amino acid residues within the peptides, evaluating the prevalence of HLA alleles, and determining the amino acid composition to construct models based on machine learning. A significant finding reaffirmed the prevailing theory that proline and glutamine are found to be prevalent in peptides associated with CD. Leveraging the composition of these peptides, the team developed ML models that reached an impressive maximum AUROC (Area Under the Receiver Operating Characteristic) of 0.99. Furthermore, they introduced an ensemble technique that merges motif-based strategies with ML models, showcasing extraordinary efficacy by accurately predicting CD-associated motifs with 100% precision on an independent dataset not previously used in the training phase.

Zammit et al. [40] applied probabilistic analysis to features identified in small bowel capsule endoscopy (SBCE) images to predict the severity of duodenal histology and differentiate between CD and non-celiac small bowel villous atrophy (SNVA). The study achieved a validation accuracy of 69.1% in both estimating the Marsh score severity and discerning between CD and SNVA. This research is pioneering in showing the capability of macroscopic features observed through SBCE to forecast the severity of the disease and to differentiate between CD and SNVA, underscoring the potential of pattern recognition in medical diagnostics.

Cao et al. [41] introduced a novel technique named DualWMDR which has been developed to pinpoint epistasis within extensive genome-wide datasets. This method marries a dual screening approach with the Weighted Multi-factor Dimensionality Reduction (WMDR) strategy. The efficacy and statistical performance of DualWMDR were scrutinized through comprehensive simulation studies that encompassed both two-locus and three-locus interactions. This involved a comparison of the effectiveness of DualWMDR against other established methodologies. Additionally, the capabilities of DualWMDR were assessed using both simulated and actual datasets, evaluating its performance relative to existing methods specifically for two- and three-locus epistatic models. The overarching goal of this research was to enhance the detection and understanding of gene interactions in genetic analyses.

Piccialli et al. [42] applied ML methods to analyze clinical data and identify the most influential features predicting patient outcomes associated with potential CD and duodenal atrophy. Feature selection methods included univariate analysis, random forests, and extremely randomized trees, while random forests, extremely randomized trees, boosted trees, and logistic regression were used for classification. They achieved high values for accuracy, specificity, and sensitivity, with the best model being the optimized boosted trees model, which had an accuracy of 0.80, a sensitivity of 0.58, and a specificity of 0.84.

Rostamkolaei et al. [43] focused on discovering biomarkers for CD through neoepitopes originating from deamidated gliadin peptides (DGPs) and fragments of tissue transglutaminase (tTG). They assessed the immunoreactivity towards these epitopes to evaluate their utility in identifying CD patients, especially those who have undergone mucosal healing. This investigation encompassed serum samples from both individuals diagnosed with CD and healthy subjects. The researchers crafted a peptide panel to examine its efficacy in pinpointing patients who have achieved mucosal healing as verified by histological assessment. Employing ML techniques, alongside Support Vector Machine (SVM) modeling, they succeeded in accurately distinguishing CD patients and those with mucosal healing. This suggests that these neoepitopes hold promise as superior biomarkers for CD, offering an advancement over current serological testing methods.

Gruver et al. [44] developed ML classifiers, which were trained by pathologists utilizing readily available AI software, to examine the histopathological characteristics of CD from immunohistochemically stained tissue samples. The practicality of this method was showcased through the analysis of paired biopsies taken before and after dietary intervention, mirroring a retrospective assessment within a clinical trial setting. When compared to traditional modified Marsh scores, this approach revealed a positive correlation with the various Marsh classifications and achieved a concordance rate exceeding 90% with the manually determined scores in the paired biopsies, despite the study’s small sample size.

The summary of the methods examined in the available literature based on clinical data analysis is shown in Table 5.

## 4. Challenges in the Standardization and Clinical Adaptation

The advancement of diagnostic methodologies for CD, particularly through imaging techniques, biomarker analysis, and computer-aided diagnostics, has undeniably enhanced the capability to detect this condition with greater accuracy and non-invasiveness. However, the path toward widespread clinical adoption of these technologies has significant challenges that must be addressed to realize their full potential in patient care [45].

1. *Variability in Imaging Techniques.* Advanced imaging methods have shown promise in identifying morphological changes associated with CD. Nonetheless, the lack of standardized imaging protocols across different healthcare facilities poses a significant challenge. The interpretation of imaging results can vary considerably due to differences in equipment, operator expertise, and diagnostic criteria. Establishing uniform imaging standards and training protocols is essential to ensure consistent and reliable diagnosis of CD across various clinical settings [46].
2. *Biomarker Analysis Specificity and Sensitivity.* While biomarker analysis offers a non-invasive avenue for CD detection, the specificity and sensitivity of these markers can be variable. The heterogeneity of CD manifestations means that no single biomarker can provide a definitive diagnosis in all cases. The challenge lies in identifying a panel of biomarkers that, when used in conjunction, offer high diagnostic accuracy. Additionally, the variability in laboratory methods and the interpretation of results can further complicate the standardization of biomarker analysis [47].
3. *Integration of Computer-Aided Diagnostics.* CAD tools harness the power of AI to analyze complex datasets and improve the diagnostic process. However, integrating these systems into clinical practice requires overcoming several hurdles. These include the need for extensive validation studies to prove their efficacy, addressing concerns related to data privacy and security, and ensuring the compatibility of these tools with existing healthcare IT infrastructure. Furthermore, the acceptance of such technologies by healthcare professionals necessitates comprehensive training and evidence of their utility in improving patients’ outcomes [1].
4. *Regulatory and Ethical Considerations* The adoption of advanced diagnostics for CD also involves navigating regulatory approvals and ethical considerations. Ensuring the safety, efficacy, and ethical use of these technologies requires rigorous clinical trials and regulatory scrutiny. Moreover, the ethical implications of widespread screening and the potential for overdiagnosis must be carefully considered, ensuring that the benefits of early detection outweigh the risks associated with unnecessary interventions.

Therefore, overcoming the challenges in the standardization and clinical adoption of diagnostics for CD is crucial for harnessing the full potential of these innovative approaches. Collaborative efforts among researchers, clinicians, regulatory bodies, and technology developers are essential to address these issues, paving the way for improved CD diagnosis and management. As we move forward, the focus must remain on developing reliable, non-invasive, and accessible diagnostic tools that can be seamlessly integrated into clinical practice, ultimately enhancing the quality of care for patients with CD.

## 5. Discussion and Conclusions

The urgent need for early and precise diagnosis of CD, a prevalent yet often underdiagnosed autoimmune disorder, underscores the pivotal role of CAD systems. In this systematic review, we highlight commonly used methods and their respective strengths and limitations. In addition, we have discussed emerging trends and advances in computer-aided diagnosis for CD, highlighting possible future directions for research and development in this important area. Our analysis is intended to contribute to the ongoing discourse on CD diagnosis and provide valuable insights for researchers, clinicians, and stakeholders committed to improving diagnostic accuracy and patient care.

The integration of advanced techniques in medical image analysis, biomarker analysis, and computer-aided models holds great potential for improving diagnostic accuracy and efficiency and reflects the ongoing efforts to enhance accuracy, efficiency, and accessibility in medical diagnostics. In medical image analysis, advancements such as the multiple-instance learning method and scale-invariant texture classification significantly contribute to the identification of characteristic changes in duodenal mucosa [16]. The application of machine learning and deep learning techniques, particularly in conjunction with endoscopic images, showcases the potential to achieve high accuracy in identifying patterns associated with CD. Biomarker analysis, including the evaluation of EmA tests and B-cell receptor repertoires, introduces non-invasive and efficient means of diagnosis. Machine learning’s role in automating the classification of EmA tests demonstrates the feasibility of rapid and unbiased analysis, potentially streamlining the diagnostic process [26]. Similarly, the identification of unique B-cell receptor motifs through machine learning offers valuable insights into potential biomarkers for CD [28]. Computer-aided models, ranging from the integration of block-wise channel squeeze-and-excitation modules to the development of Hierarchical Medical Image Classification (HMIC) methods, underscore the importance of innovation in deep learning architectures [17]. The ability to adaptively recalibrate image features and the hierarchical analysis of medical images provide a robust foundation for accurate celiac disease detection. Clinical data analysis, including predictive modeling and risk factor identification, enhances our understanding of the associations between demographic, clinical, and histopathological variables in CD [21,42]. The use of ML in predicting epitopes associated with celiac disease and assessing severity through macroscopic features demonstrates the potential for computational tools to contribute to a more nuanced understanding of the disease.

**Figure 5:**
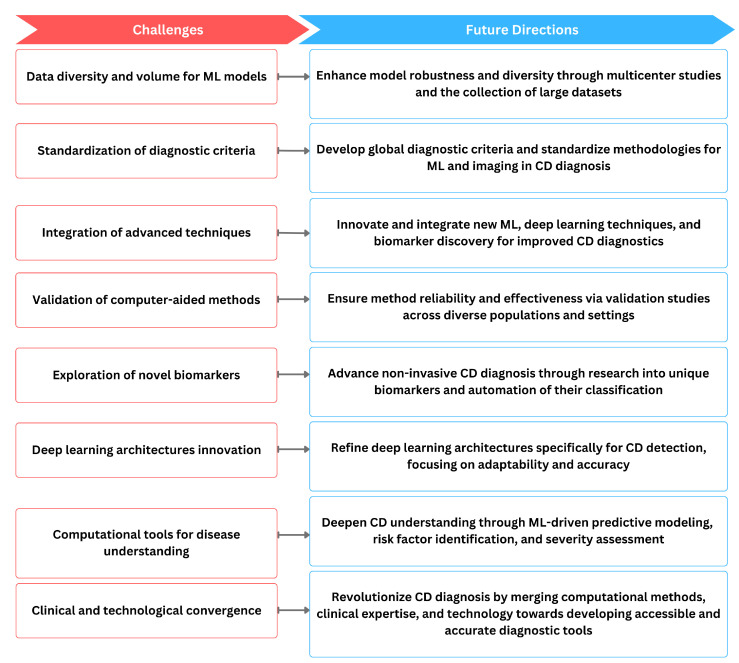
The relationship of current challenges in computer-aided diagnosis methods of CD and possible future directions.

However, common ML challenges such as the need for large, diverse datasets for robust model training and validation persist in this field as well as shown in Figure 5. Standardization of the diagnostic criteria and methodologies across studies is essential for ensuring the generalizability of computer-aided methods in clinical practice. As technology continues to evolve, there is a clear trajectory toward more sophisticated and accurate diagnostic tools for CD. The ongoing refinement of machine learning algorithms, deep learning architectures, and the exploration of novel biomarkers contribute to the overall progress in the field. The continuous validation of these methods in diverse patient populations and clinical settings is imperative to ensure their reliability and effectiveness. Ultimately, the convergence of these computer-aided methods has the potential to revolutionize CD diagnosis, providing clinicians with powerful tools to enhance patient care. Nevertheless, several practical challenges that could affect the deployment and efficacy of these technologies remain. Interdisciplinary collaboration is crucial for addressing these challenges, as it brings together expertise from gastroenterology, immunology, computer science, and bioinformatics. This collaborative approach is vital for tackling issues such as the heterogeneity of clinical presentations and the variability in the quality of medical imaging across different centers. Moreover, while CAD can significantly reduce the time and cost associated with diagnosing CD, it also raises concerns about the potential for over-reliance on technology at the expense of clinical expertise. To mitigate this, it is essential to develop guidelines for the judicious use of CAD, ensuring that it complements rather than replaces traditional diagnostic methods. Furthermore, there is a need for comprehensive regulatory frameworks to oversee the clinical implementation of CAD systems, ensuring they meet safety and efficacy standards before widespread adoption. Addressing these aspects will be crucial for realizing the full potential of CAD in revolutionizing the diagnosis of celiac disease and potentially other autoimmune disorders. We hope this review will provide valuable insights for future investigations and implementation studies, fostering a path toward a more accessible, accurate, and timely diagnosis of CD.

In conclusion, the prospects for the integration of computer-aided diagnosis in the treatment of CD are promising and developing rapidly. Future developments in CAD are likely to focus on improving the precision and accuracy of diagnostic algorithms by incorporating advanced ML techniques. These aim to analyze complex patterns in diagnostic data more effectively, potentially reducing the need for invasive procedures such as biopsies. In addition, the integration of CAD systems into electronic health records could streamline the diagnostic process and enable faster and more accurate diagnoses, which are crucial for the timely treatment of CD. In addition, there is strong interest in the development of portable diagnostic tools that could utilize CAD to make screening and early detection more accessible in primary care. These advances will require continued collaboration between researchers, clinicians, and technicians to ensure that the benefits of CAD systems are realized in various healthcare settings. These systems must be rigorously tested in clinical trials to ensure their efficacy and safety and to promote wider adoption and implementation in clinical practice.

## Figures and Tables

**Table 1:** Summary of database and search terms.

Database	Search Terms
Science Citation Index Expanded	computer aided, cad, deep learning, machine learning, artificial intelligence, celiac disease, celiac sprue, segmentation, detection, classification, identification, evaluation, diagnosis
Emerging Sources Citation Index	
MEDLINE	
Data Citation Index	
Conference Proceedings Citation Index	
KCI-Korean Journal Database	
BIOSIS Citation Index	
Derwent Innovations Index	
SciELO Citation Index	
Russian Science Citation Index	

**Table 2:** Summary of methods based on medical image analysis.

Authors	Method	Dataset	Modality	Results
Syed et al. [19]	CNN	3118 images	Biopsy	ACC: 93.4%
Tyagi et al. [25]	DeGPR	55 images	Biopsy	ACC: 87.7%
Faust et al. [20]	SVM	91 images	Biopsy	ACC: 98.53%
Syed et al. [18]	CNN	461 images	Endoscopic	ACC: 98%
Garbaz et al. [23]	CNN	50000 images	Endoscopic	ACC: 98.5%
Hegenbart et al. [16]	DT-CWT	612 images	Endoscopic	OCR: 84.7%
Saken et al. [15]	DWT-LBP-cubic SVM	330 images	Endoscopic	ACC: 94.79%
Sali et al. [21]	CNN	120 images	Histological	ACC:
				MarshIIIc:
				90.61%
Elmes et al. [24]	CNN	30 images	Histological	ACC: 76.6%
Kowsari et al. [17]	DCNN, HMIC	461 images	Histological	ACC:
				(DCNN) 82.95%
				(HMIC) 88.01%
DiPalma et al. [22]	KD	1364 patients	Histological	ACC: 85.74%

**Table 3:** Summary of methods based on biomaker analysis.

Authors	Method	Dataset	Modality	Results
Caetano dos Santos et al. [26]	SVM	2597 images	IgA-class EmA	ACC: 96.8%
Magazzù et al. [27]	fuzzy CNN	20,519 images	IgA-class EmA	ACC: 99%
Shemesh et al. [28]	MIL	100 patients	Immune repertoire	F1 score: 85%

**Table 4:** Summary of methods based on computer-aided models.

Authors	Method	Dataset	Modality	Results
Khan et al. [30]	CNN	290 images	Biopsy	ACC: 49%
Koh et al. [35]	Feature engineering	91 images	Biopsy	ACC: 88.89%
	and ML			
Molder et al. [32]	CNN	82 images	Endoscopy	ACC: 98.24%
Stoleru et al. [4]	Linear SVM	109 films	Endoscopy	ACC: 94.1%
Maleki et al. [38]	CNN	426 images	Histological	ACC: 89.3%
Wimmer et al. [37]	CNN	1045 images	WLI	ACC: 85.6%
		616 images	NBI	ACC: 88.3%
Wang et al. [29]	BCSE	2140 images	VCE	ACC: 95.94%
Koh et al. [31]	SVM RBF	13 patients	VCE	ACC: 86.47%
Vicnesh et al. [33]	SVM RBF	37 patients	VCE	ACC: 89.82%
Zammit et al. [34]	MLA	63 videos	VCE	α^1^ > 0.8

^1^ Krippendorff’s alpha value.

**Table 5:** Summary of methods based on clinical data analysis.

Authors	Method	Dataset	Modality	Results
Tomer et al. [39]	ML	1310 peptides	-	ACC: 98%
Zammit et al. [40]	ML	72 patients	SBCE	ACC: 75.3%
Cao et al. [41]	DualWMDR	435,604 SNP	Genome-wide	PER < 0.5
Piccialli et al. [42]	ML	340 patients	Serology, biopsy	ACC: >75%
Rostamkolaei et al. [43]	ML	169 patients	Serology	ACC: 99%
Gruver et al. [44]	ML	116 patients	Histological	Correlation: >90%

## Data Availability

No new data were created or analyzed in this study.

## Abbreviations

The following abbreviations are used in this manuscript:

anti-tTG	Anti-Tissue Transglutaminase
anti-EMA	Anti-Endomysial Antibodies
ACC	Accuracy
AUROC	Area Under the Receiver Operating Characteristic
BCR	B-cell Receptor
BCSE	Block-wise Channel Squeeze-and-Excitation
CAD	Computer-Aided Diagnosis
CD	Celiac Disease
CDSS	Clinical Decision Support System
CNN	Convolutional Neural Network
DCNN	Deep Convolutional Neural Network
DEGPR	Deep Guided Posterior Regularization
DGP	Deamidated Gliadin Peptide
DT-CWT	Dual-Tree Complex Wavelet Transform
DWT	Discrete Wavelet Transform
EE	Environmental Enteropathy
EED	Environmental Enteric Dysfunction
grad-CAMs	gradient-weighted Class Activation Maps
HLA	Human Leukocyte Antigen
HMIC	Hierarchical Medical Image classification
KD	Knowledge Distillation
LOOCV	Leave-One-Out Cross-Validation
LTP	Local Ternary Pattern
LTPs	Local Ternary Patterns
MIL	Multiple Instance Learning
NBI	Narrow-band Imaging
NCD	Non-Celiac Disease
OCR	Overall Classification Rate
PER	Prediction Error Rate
POCT	Point-of-Care Testing
PSO	Particle Swarm Optimization
RGB	Red Green Blue
SBCE	Small Bowel Capsule Endoscopy
SE	Squeeze and Excitation
SNP	Single Nucleotide Polymorphisms
SNVA	Small Bowel Villous Atrophy
SPT	Steerable Pyramid Transform
SVM	Support Vector Machine
VA	Villous Atrophy
VCE	Video Capsule Endoscopy
WLI	White-light Imaging
WMDR	Weighted Multi-factor Dimensionality Reduction
WSI	Whole Slide Images
